# Not ‘Inactive’ After All: Cardiotoxic Mechanisms of Catecholamine Metabolism by Monoamine Oxidase

**DOI:** 10.1007/s12012-025-10021-7

**Published:** 2025-06-10

**Authors:** Rachel M. Crawford, Ethan J. Anderson

**Affiliations:** 1 Department of Pharmaceutical Sciences & Experimental Therapeutics, College of Pharmacy, University of Iowa, 180 S Grand Avenue, Iowa City, IA 52242 USA; 2 Fraternal Order of Eagles Diabetes Research Center, University of Iowa, Iowa City, IA 52242 USA; 3 Abboud Cardiovascular Research Center, University of Iowa, Iowa City, IA 52242 USA

**Keywords:** Monoamine oxidase, Cardiotoxicity, Hydrogen peroxide, Ammonia, Catecholaldehyde, Mitochondria, Cardiovascular disease

## Abstract

Monoamine oxidase (MAO) helps regulate catecholaminergic signaling via metabolism of neurotransmitters epinephrine, norepinephrine, and dopamine—in turn producing the metabolites hydrogen peroxide (H_2_O_2_), ammonia (NH_4_^+^), and corresponding catecholaldehydes. While MAO has been a key facet of neuroscience and mood disorder research for > 60 years, MAO-generated metabolites have been largely overlooked until recently when reports have begun to illustrate the reactivity of these metabolites and their pathogenic contributions to disease (e.g., inflammation, fibrosis, cell death). These findings have extended MAO’s biological relevance beyond the brain and, most notably, to the heart, where a large and growing body of literature clearly indicates a pathophysiologic role for MAO-mediated catecholamine metabolism in heart disease. Herein, we discuss the evidence connecting MAO to various cardiac injuries and disorders, as well as describe the known cardiotoxicity associated with MAO’s reactive metabolites, specifically in connection to cardiac pathophysiology. Potential therapeutic strategies for targeting MAO and its metabolites to prevent and treat heart disease are also discussed, and important knowledge gaps highlighted.

**Graphical Abstract:**

Created using biorender.com

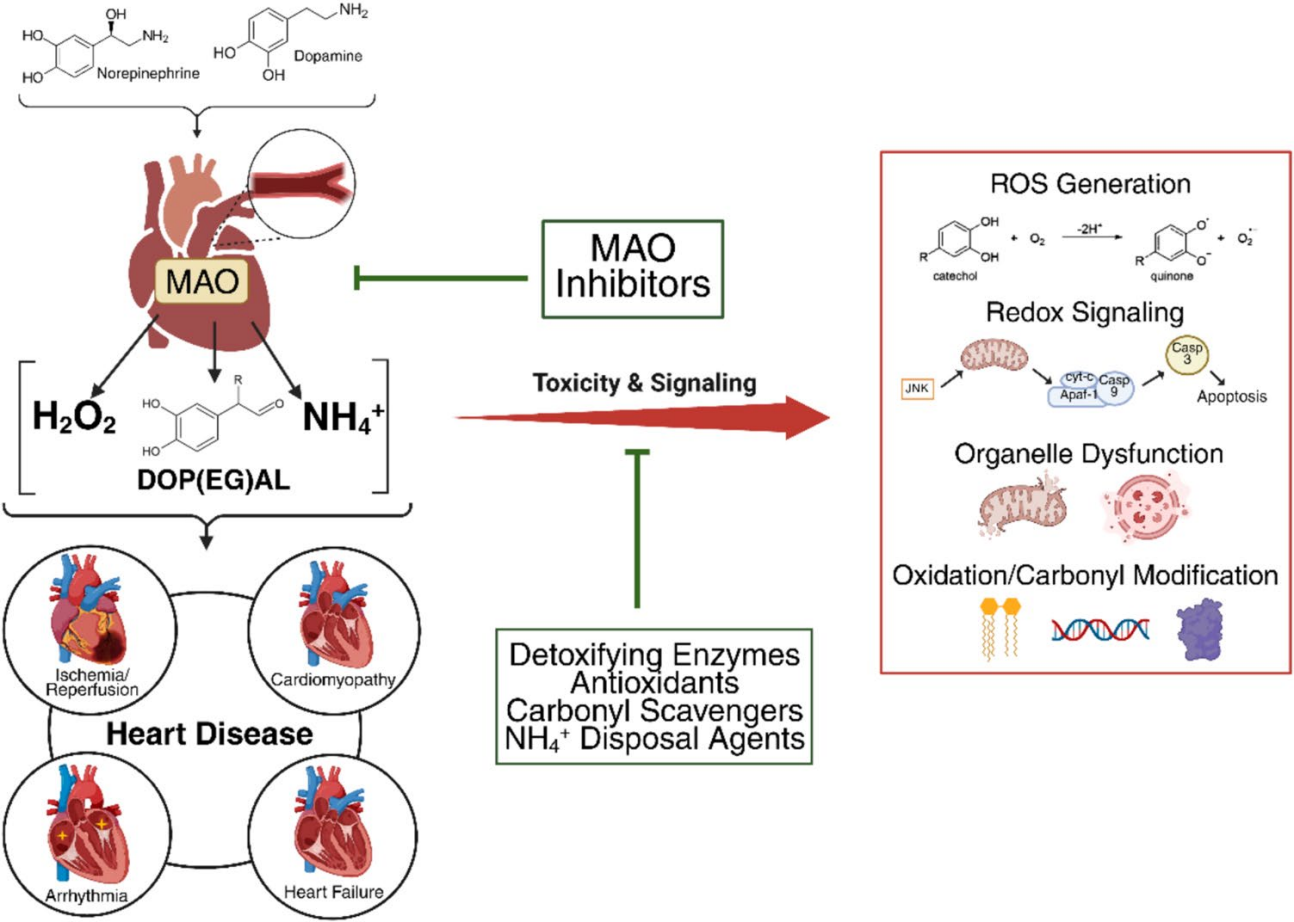

## Introduction

Despite intensive research for > 75 years, the role and function of monoamine oxidase (MAO) in health and disease is only beginning to be understood and new discoveries seem to emerge every year. MAO catalyzes the oxidative deamination of monoamine neurotransmitters and in the process, exerts tight control over autonomic physiology and most bodily functions. All products formed by MAO—hydrogen peroxide (H_2_O_2_), ammonia (NH_4_^+^), and highly reactive biogenic aldehydes—are biologically active and it is noteworthy that most modern pharmacology textbooks [1, 2] have failed to catch up with contemporary research that has increasingly documented pathophysiological effects caused by these products. The specific pathways and mechanisms by which these products might be contributing to cardiovascular disease are the focus of this review.

MAO’s importance as a drug target for mood disorders was recognized in early studies owing to the striking effects of MAO inhibition on regulating brain concentrations of serotonin (5-HT), dopamine (DA), epinephrine (Epi), and norepinephrine (NE) [3]. This led to MAO inhibitors (MAOIs) being among the first pharmacotherapies for depression and Parkinson’s disease (PD). These findings firmly established the clinical relevance of MAO and prompted subsequent research to improve our understanding of the enzyme. It is now known that MAO is a flavoenzyme anchored to the outer mitochondrial membrane and exists in two isoforms, MAO-A and -B. MAO is found in almost all human tissues, however expression and localization vary between isoforms. MAO-A is the predominant isoform in myocardial tissue and catecholaminergic neurons while MAO-B is localized to astrocytes and serotonergic neurons. MAO isoforms also differ in substrate specificity with MAO-A preferentially metabolizing 5-HT and NE, MAO-B having high affinity for benzylamine and phenylethylamine, and both isoforms oxidizing tyramine and DA. Recent findings suggest a more limited role in DA regulation by MAO-B [4]. Owing to the difference in substrate specificity, selective inhibitors have been developed for each isoform. The MAOIs clorgiline and moclobemide target MAO-A while MAO-B is inhibited by selegiline, rasagiline, and safinamide.

Over the last few decades, researchers have begun to closely examine the metabolic byproducts formed via MAO during the oxidative deamination of catecholamines. Two major developments that spurred this new focus were: (1) the concept of redox signaling that emerged in the late 1980’s and alerted researchers to the importance of enzymes that generate reactive oxygen species (ROS), such as MAO [5], and (2) the discovery and increasing evidence that aldehyde metabolites such as 3,4-dihydroxyphenylacetaldehyde (DOPAL) and 3,4-dihydroxyphenylglycolaldehyde (DOPEGAL), formed via MAO metabolism of DA and NE, respectively, has pathogenic effects in the nervous, cardiovascular, and immune systems (reviewed in [6–10]). These developments sparked many new studies directed at examining not just the canonical role of MAO in regulating catecholaminergic tone, but also the potential role of MAO byproducts and their contributions to disease.

## Cardiac Disorders Associated with MAO

Experimental and clinical work over the past 20 years has firmly established that MAO in the myocardium itself is linked with development of many cardiac disorders and disease [11, 12]. MAO is the enzyme solely responsible for monoamine metabolism in the heart as catechol-o-methyltransferase (COMT), while abundant in CNS, is not expressed in human cardiomyocytes [13]. The importance of catecholamine metabolism to proper cardiac electromechanical function has recently been highlighted by in vivo and post-mortem studies showing that patients with synucleinopathies (e.g., Lewy body diseases, PD) have sharply diminished cardiac NE levels compared with normal patients [14, 15]. In fact, the pathogenesis of most cardiac injury and diseases involve catecholaminergic signaling [16–18] and oxidative stress [19–21] in the heart. It is highly plausible that MAO may be the common unifying thread in the pathophysiological process, strongly suggesting a mechanistic role for MAO in the pathogenesis of cardiovascular diseases. In the following sections we briefly summarize the current literature connecting MAO to cardiac injury/disease, discuss the known cytotoxic effects of MAO-mediated catecholamine metabolites, and examine how these toxic effects have been shown to contribute to cardiac disease. It is important to note here that MAO metabolizes serotonin at high rates as well, and thus it cannot be ruled out that many of the effects seen with MAO inhibition may be indirectly attributable to increased serotonergic activation [22–24]. These indirect effects of MAO inhibition in the context of global physiologic signaling and myocardial function is beyond the scope of this review. Emphasis in sections below will be placed on studies that used pharmacologic and genetic inhibition of MAO in non-neuronal cell-specific models to elucidate the enzyme’s role in cardiac disorders. Table 1 summarizes original research articles attributing MAO to the pathophysiology of several cardiac injuries/diseases.

**Table 1:** Summary of studies investigating cardiac disorders related to MAO

Cardiac disease/injury	Subject model	Treatment groups	Key findings	References
Ischemia/Reperfusion	Rats with coronary artery suture	Non-treated controls Pargyline treatment Clorgiline treatment	MAO inhibition improved myocardial structure, reduced pro-apoptotic protein expression, reduced lipid peroxidation, and reduced number of pro-inflammatory cells associated with non-treated I/R rats	[26] Bianchi, et al. *Circulation*, 2005
Ischemia/Reperfusion	Ex vivo mouse hearts undergone I/R via Langendorff perfusion	WT MAO-B KO	Hearts from MAO-B KO mice experienced smaller infarct sizes following I/R and generated lower amounts of ROS	[27] Heger, et al. *Free Radic Biol Med*, 2021
Ischemia/Reperfusion	Mice undergone coronary occlusion	WT MAO-B KO	Compared I/R effects on male vs. female mice and found that female mice were significantly more protected from myocardial damage compared to males. For this reason, MAO-B KO reduced infarct size only in male mice	[28] Heger, et al. *Int J Mol Sci*, 2023
Ischemia/Reperfusion	Rats undergone coronary artery suture	Non-treated controls Pargyline treatment	MAO inhibition reduced markers for oxidative stress and cardiomyocyte injury compared to non-treated controls	[29] Inagaki, et al. *Free Radic Res*, 2016
Ischemia/Reperfusion	Ex vivo mouse hearts undergone I/R via Langendorff perfusion	Non-treated controls Pargyline treatment	MAO inhibition reduced tropomyosin oxidation, reduced pro-apoptotic markers, and improved recovery compared to non-treated hearts	[30] Carpi, et al. *Biochem Biophys Acta*, 2009
Ischemia/Reperfusion	Rats undergone coronary artery suture	Non-treated controls Moclobemide treatment	Moclobemide-treated rats saw reduced expression of stress-response proteins and reduced inflammation in hearts compared to non-treated	[31] Vuohelainen, et al. *Interact Cardiovasc Thorac Surg*, 2015
Ischemia/Reperfusion	Rats undergone coronary artery suture	Non-treated controls Rasagiline treatment	Rasagiline-treated rats had preserved cardiac functional parameters, prevented collagen deposition, and reduced cell death compared to non-treated	[32] Varela, et al. *ESC Heart Fail*, 2017
Ischemia/Reperfusion	Ex vivo rat hearts undergone I/R via Langendorff perfusion	Non-treated controls Ischemic Preconditioning (IPC) IPC + clorgiline (IPC-C) IPC + pargyline (IPC-P)	Improved functional recovery associated with IPC group was enhanced even further with the additional clorgiline and pargyline treatment. Infarct size was reduced with IPC groups and this improvement was not changed by the addition of MAO inhibitors	[33] Danila, et al. *Can J Physiol Pharmacol*, 2015
Ischemia/Reperfusion	Human ventricle tissue	Non-failing controls (NF) Non-ischemic heart failure (HF) Ischemic heart failure (IHF)	MAO-A/B activity and expression were elevated in ventricles of IHF hearts along with oxidative stress markers. Found strong correlation between increased MAO-A activity, increased actin oxidation, and impaired cardiac function of IHF hearts	[34] Manni, et al. *Oxid Med Cell Longev*, 2016
Ischemia/Reperfusion	Human mammary artery tissue	Untreated Clorgiline Moclobemide Selegiline	Endothelial-dependent relaxation was improved in human mammary artery tissue following MAOI treatment compared to untreated	[35] Lighezan, et al. *Can J Physiol Pharmacol*, 2016
Aging	Rats	Aged 1, 3, 6, & 24 months	Age-dependent increase in MAO-A activity and MAO-dependent H_2_O_2_ production	[37] Maurel, et al. *Am J Physiol Heart Circ Physiol*, 2003
Aging	Rats	Aged 3–4, 13–15, & 24 months	Age-dependent increase in MAO activity	[38] Savitha, et al. *Eur J Pharmacol*, 2007
Aging	Culture cardiomyoblasts Primary cardiomyocytes	Tyramine ± clorgiline NE ± clorgiline	Activation of MAO by tyramine and NE increased cell senescence; clorgiline treatment mitigated this effect	[39] Manzella, et al. *Aging Cell*, 2018
Pressure Overload	Mice undergone transverse aortic constriction	WT Dominant negative MAO-A (MAO-A^neo^) or Non-treated controls Clorgiline treatment	Clorgiline treatment reduced oxidative stress and apoptosis in heart and prevented LV dilation and dysfunction in mice 6wk after TAC compared to non-treated. Similar results were observed in MAO-A^neo^ mice 9wk after TAC compared to WT	[42] Kaludercic, et al. *Circ Res*, 2010
Pressure Overload	Mice undergone transverse aortic constriction	WT MAO-B KO	MAO-B KO mice had reduced LV remodeling and dysfunction compared to WT mice. This was associated with reduced oxidative stress markers and improved mitochondrial function in MAO-B KO group	[43] Kaludercic, et al. *Antioxid Redox Signal*, 2014
Pressure Overload	Mice undergone ascendant aortic banding	WT MAO-A KO	Observed increased cardiac hypertrophy in MAO-A KO mice compared to WT. This was associated with elevated 5-HT levels and 5-HT_2A_ receptor overexpression. Treatment with 5-HT receptor antagonist reduced these effects	[44] Lairez, et al. *J Mol Cell Cardiol*, 2009
RV Heart Failure	Human RV tissue samples and Rats with Sugen5416 and hypoxia-induced pulmonary arterial hypertension (PAH)	Human: Patients with PAH Patients without PAH Rat: Non-treated controls Clorgiline treatment	MAO-A expression was elevated in RV of patients with PAH compared to patients without. This was consistent in PAH rat models. Inhibition of MAO-A with clorgiline reduced stiffness, improved relaxation, and reversed hypertrophy in RV compared to non-treated rats	[46] Sun, et al. *Am J Respir Cell Mol Biol*, 2021
RV Heart Failure	Mice undergone pulmonary artery banding	WT MAO-B KO	MAO-B KO mice saw reduced ROS levels and reduced kinase expression associated with hypertrophy compared to WT mice. WT mice with pulmonary artery banding experienced significant RV dilated hypertrophy which was prevented in MAO-B KO group	[47] Brosinsky, et al. *Int J Mol Sci*, 2024
Arrhythmia	Human atrial tissue	Not Applicable	Found strong association between increased MAO expression in cardiac tissue and increased risk of post-operative atrial fibrillation in human patients	[49] Anderson, et al. *J Am Heart Assoc*, 2014
Arrhythmia	Patients treated for depression and Mice undergone catecholamine stress-induced tachycardia	Patients: MAOI treatment SSRI treatment Mice: WT MAO-A deficient (MAO-A^def^)	Found that patients treated with MAOI had reduced risk and incidence of cardiac arrhythmias compared to SSRI-treated patients. MAO-A^def^ mice were more protected from incidence of catecholamine-induce arrhythmias compared to WT. This was associated with reduced ROS production, reduced protein oxidation, and improved calcium handling in MAO-A^def^ mice compared to WT	[50] Shi, et al. *Cardiovasc Res*, 2024
Diabetes/Obesity	Human atrial tissue	Non-diabetic control patients Diabetic patients	Found elevated MAO-A/B activity and expression, reduced ALDH activity, increased levels of catechol-modified protein adducts, and impaired mitochondrial oxidative phosphorylation in diabetic tissue samples compared to non-diabetic samples	[54] Nelson, et al. *Antioxid Redox Signal*, 2021
Diabetes/Obesity	Human atrial tissue	Healthy Controls; Coronary Heart Disease (CHD); CHD with diabetes (CHD-DM)	Impaired mitochondrial respiration in CHD samples was exacerbated in CHD-DM group. Significantly higher MAO-dependent ROS production in CHD and CHD-DM groups compared to controls	[55] Duicu, et al. *Oxid Med Cell Longev*, 2016
Diabetes/Obesity	STZ-induced diabetic rats	Non-treated controls Clorgiline treatment	STZ-induced diabetes was associated with increased MAO-A activity, oxidative stress markers, apoptosis, and fibrosis. Clorgiline treatment attenuated these effects and improved cardiac functional parameters in rats	[56] Umbarkar, et al. *Free Radic Biol Med*, 2015
Diabetes/Obesity	STZ-induced diabetic mice	Non-treated controls Pargyline treatment	Pargyline treatment reduced oxidative stress, ER stress, and collagen deposition seen in non-treated STZ-induced diabetic mice. MAO inhibition also improved diastolic function	[57] Deshwal, et al. *Cell Death Differ*, 2018
Diabetes/Obesity	STZ-induced diabetic rats	Non-treated controls Clorgiline treatment Selegiline treatment	Elevated MAO-A/B expression in heart and aorta of diabetic rats was associated with increased ROS and impaired endothelial-dependent relaxation. Inhibition of MAO-A/B reduced ROS and improved endothelial relaxation	[58] Sturza, et al. *Can J Physiol Pharmacol*, 2015
Diabetes/Obesity	Diet-induced obese rats	Non-treated controls Metformin treatment Clorgiline treatment Selegiline treatment	Elevated MAO-A/B activity and oxidative stress was associated with obesity in rats. Treatment with metformin and MAO inhibitors reduced oxidative stress in obese rats. Importantly, metformin found to down regulate MAO expression in obese cardiac tissue	[59] Merce, et al. *Mol Cell Biochem*, 2023

### Ischemia/Reperfusion Injury

Some of the earliest work connecting MAO to cardiac dysfunction was in models of ischemia/reperfusion (I/R) injury. Both catecholaminergic tone and ROS surge during reperfusion [25] and MAO mechanistically links these pathways. One study exploring this connection used coronary artery suture to induce I/R injury in rats with three treatment groups: no treatment, MAO-A inhibition (clorgiline), or nonselective MAO inhibition (pargyline). Clorgiline and pargyline treatments reduced myocardial structural damage, reduced cell death and pro-apoptotic protein expression, and mitigated oxidative stress following I/R injury compared to non-treated rats [26]. These findings were supported by subsequent studies in a variety of I/R injury models documenting protective effects with MAO-A/B inhibition [27–31]. In these later studies, MAO inhibition also reduced infarct size and improved myocardial function. One group found that MAO–B inhibition in the days following I/R injury, rather than before or during injury, was still effective in improving myocardial recovery in rats, suggesting that persistent MAO inhibition post-infarction may be effective at mitigating adverse cardiac remodeling [32]. Authors of these studies mostly attributed the cardioprotective mechanisms of MAO inhibition to be driven by decreased ROS generation, although one group also suggested that improved contractility observed with MAOI treatment may be due to increased catecholamine availability to the stunned myocardium [33].

MAO-mediated stress associated with I/R that has been demonstrated in animal models was also observed in human heart tissue ex vivo. Ventricular myocardial samples were taken from three groups of patients: heart failure secondary to ischemia, non-ischemic heart failure, and non-failing control hearts. In these experiments MAO-A/B activity and expression were elevated only in the ischemic hearts, along with oxidative stress markers [34]. Other work found that mammary artery sections of coronary heart disease patients had improved relaxation following treatment with MAO-A and MAO-B inhibitors compared to untreated [35]. This suggests that MAO inhibition could prevent I/R injury by improving endothelial function in coronary artery disease, a common cause of cardiac ischemia.

### Aging

Aging is invariably accompanied by myocardial weakening and fibrosis, making it a dominant risk factor for developing cardiomyopathy [36]. Several studies found that cardiac MAO activity was positively correlated with aging in rat models [37, 38]. One study found a 7.5-fold increase in MAO-A activity in 24 month-old rats compared with 1 month-old [37]. An in vitro ‘cellular aging’ model using low concentrations of tyramine and NE over days in culture determined that chronic MAO-A activation in cardiomyoblasts and primary cardiomyocytes led to increased cellular senescence characterized by increased expression of cell cycle inhibitors, enhanced DNA damage response, and cell flattening [39]. These changes were mitigated with clorgiline, indicating that MAO-A is not only associated with cardiac aging but may play an active part in the molecular mechanisms underlying age-related cardiac dysfunction (e.g., senescence).

### Pressure Overload

Pressure overload imposes significant stress on the left ventricle and leads to left ventricular (LV) remodeling, hypertrophy, and heart failure [40, 41]. To compensate for the high afterload, sympathetic signaling increases and, likely due to increased substrate availability, MAO expression and activity also increase in the heart. Studies of pressure overload in mouse models have shown that MAO-A/B-dependent catecholamine catabolism increases in myocardium during the adaptation to the stress [42, 43]. Importantly, inhibiting MAO in pressure overload models has been shown to benefit cardiac structure and function in these studies. In one report using echocardiography and pressure–volume loop recordings, both pharmacological (clorgiline-treated) and genetic (dominant negative mutation) inhibition of MAO-A in mouse heart reduced LV dilation and preserved LV function 6 weeks after transverse aortic constriction (TAC) compared with untreated control mice [42]. Another study observed similar findings with MAO-B knockout mice 9 weeks after TAC [43]. In contrast, it was also reported that whole body MAO-A knockout mice exhibit increased hypertrophy with ascendant aortic banding due to increased serotonin receptor activation [44]. Taken together, these findings suggest that while reduced MAO activity in the myocardium itself may be beneficial in pressure overload, the potentially deleterious impact of global MAO inhibition (i.e., in the brain) must also be taken into consideration in the context of therapy.

### Right Ventricular Heart Failure

Right ventricular (RV) heart failure often accompanies lung disease and is a leading cause of pulmonary hypertension [45]. MAO-A/B activity has been found to increase in the LV as well as the RV during cardiac injury suggesting a possible role for MAO during RV stress [34]. Recent work addressing this possible connection used pulmonary artery banding in rats to induce pulmonary hypertension with and without clorgiline administration. Clorgiline treatment reduced stiffness, improved relaxation, and mitigated hypertrophy in the RV compared with untreated rats [46]. A similar study of pulmonary artery banding was performed in WT and MAO-B knockout mice. This showed improved RV structure and function and decreased oxidative stress and hypertrophy-associated kinase activity in MAO-B knockout mice compared with WT [47].

### Arrhythmia

Oxidative stress and deranged catecholaminergic signaling have long been associated with arrhythmias, therefore MAO may be an obvious therapeutic target of interest for this disease [48]. One study used right atrial tissue samples from patients undergoing cardiac surgery and found a strong correlation between increased MAO activity in the tissue and incidence of post-operative atrial fibrillation [49]. The clinical relevance of MAO-A in arrhythmia was further demonstrated by work showing that patients taking MAOIs for mood disorders were more protected from cardiac arrhythmias compared to age-matched patients taking SSRIs [50]. Using a mouse model, the authors also showed that MAO-A deficient mice were protected from epinephrine/caffeine induced arrhythmogenesis compared with WT, and this was determined to be due in part to decreases in oxidized CaMKII and calcium spark frequency in the MAO-A deficient mouse hearts.

### Obesity/Diabetes

Metabolic disorders such as obesity and Type 2 diabetes are known to increase catecholamine levels in the heart [51–53]. Studies of MAO in human atrial appendage samples found increased expression and activity of both MAO-A/B isoforms in obese patients with diabetes compared with obese non-diabetes patients [54]. Another group performed similar measurements in atrial appendage samples from patients with coronary heart disease (CHD), with (CHD-DM) and without diabetes, compared with no diabetes or CHD. Expression of MAO was comparable among all three groups. However MAO-dependent ROS was elevated in the CHD and CHD-DM groups compared with controls, indicating MAO may act as a major source of ROS in patients with cardiometabolic disease [55]. Both of these studies also found MAO-dependent impairments to mitochondrial oxidative phosphorylation (OxPHOS) in heart samples from diabetes patients [54, 55]. These findings were consistent with animal model studies where MAO expression and activity were elevated in both streptozotocin (STZ)-induced diabetes [56–58] and diet-induced obesity [59]. In one of these studies, treatment with clorgiline diminished oxidative stress markers, reduced cellular apoptosis and fibrosis, and improved cardiac contractile function compared to non-treated STZ-induced diabetic rats [56]. Similarly, pargyline treatment reduced cardiac inflammation and improved diastolic function in STZ-induced diabetic mice compared to the non-treated group [57].

Important limitations to the studies above that should be noted include the fact that increased 5-HT levels are known to suppress NE neuronal firing [22, 23]. While this effect has not yet been observed in the heart it is possible that inhibiting MAO-A prevents 5-HT metabolism, increasing 5-HT levels and suppressing NE release in the heart. Therefore benefits of clorgiline treatment observed in the above studies may be due to reduced β-adrenergic activation rather than reduced MAO-mediated metabolites. Additionally, while nearly every paper described above attributes the benefits of reduced MAO activity to reduced metabolite generation, these effects may also be due to elevated levels of signaling monoamines like 5-HT, NE, and DA.

## Catecholamine Metabolites are Biologically Active and Contribute to Homeostasis and Disease

**Fig. 1:**
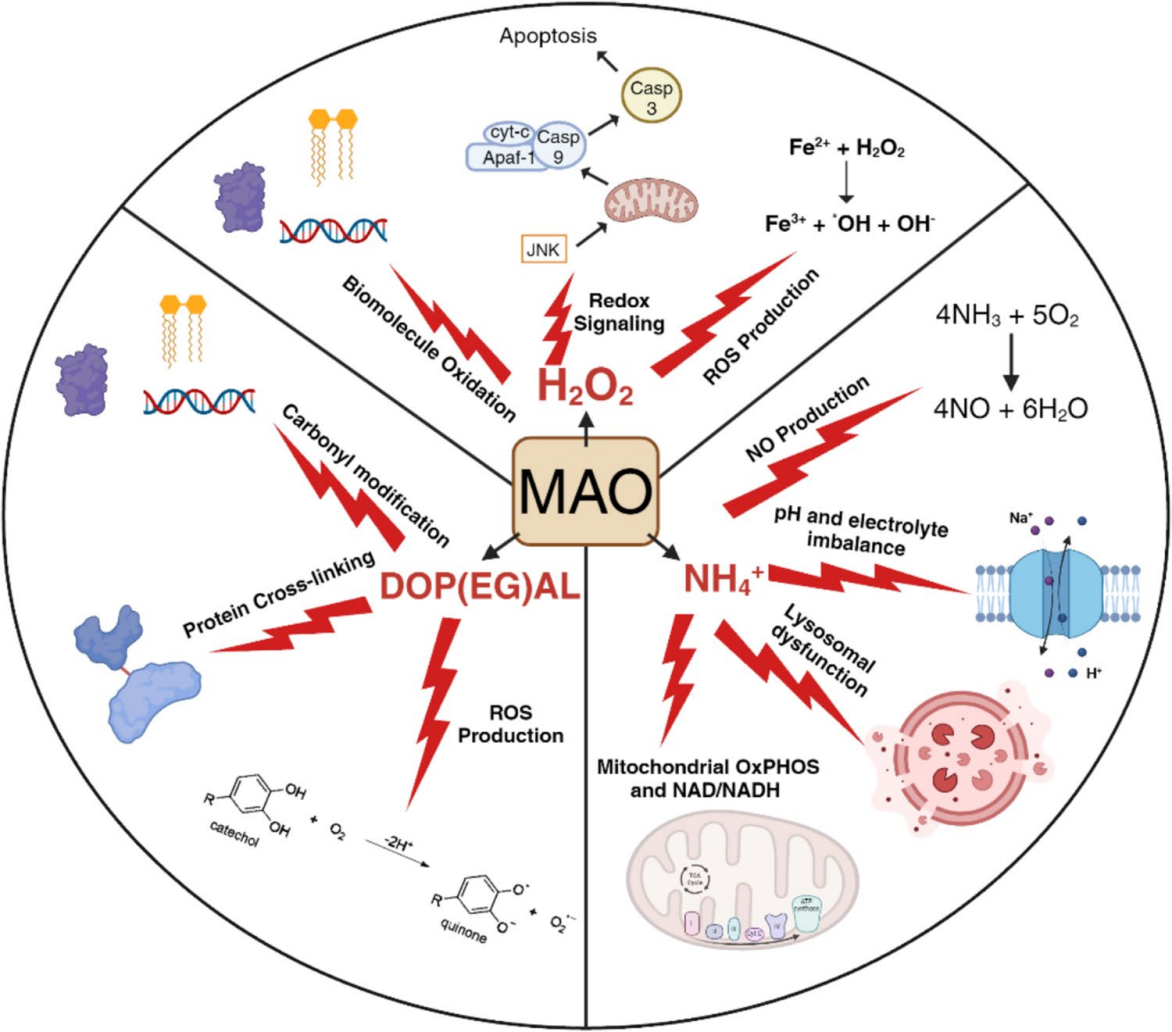
Pathogenicity of catecholamine metabolites mediated by MAO in cells. H_2_O_2_, NH_4_^+^, and DOP(EG)AL are generated via MAO’s oxidative deamination of the catecholamines epinephrine, norepinephrine, and dopamine. At excess levels these metabolites cause severe damage and dysfunction in the cell. High H_2_O_2_ levels cause changes to redox signaling, increased biomolecule oxidation, and increased production of other ROS. High DOP(EG)AL levels also result in biomolecule oxidation and ROS production as well as protein conjugation and cross-linking. Elevated NH_4_^+^ levels cause NO production, membrane depolarization, pH imbalance, and mitochondrial energetic inhibition. Created using bioRender.com

As described above, oxidative deamination of catecholamines by MAO generates catecholaldehydes, H_2_O_2,_ and NH_4_^+^ as byproducts. Since its discovery, a majority of research on MAO has focused on its ability to inactivate and remove neurotransmitters thus regulating their signaling. A special interest in the metabolites formed by MAO has gained traction due to emerging new evidence that these metabolites all participate to varying extent in cellular homeostasis, function, and disease. In this section, we provide a current summary of the known biological roles of each metabolite individually and discuss their potential importance in physiological function and disease (Fig. 1).

### H_2_O_2_

Since its discovery and characterization nearly two centuries ago, H_2_O_2_ has largely been considered a toxic molecule and its sole physiological function limited to killing pathogens as part of the immune system. Work in the 1970’s established that nearly all cell types generate steady state levels of H_2_O_2,_ and this ushered in a new era where biochemists began to appreciate a more significant and nuanced role for this molecule in the cell [60, 61]. It is now understood that H_2_O_2_ is a key molecule in redox signaling through networks of protein oxidation within the cellular environment. As such, H_2_O_2_ is important for regulating a number of cellular processes including cell growth, stress response, and apoptosis [62–64]. Because of H_2_O_2_’s dual role in cellular signaling and as agent of toxicity, cells tightly regulate its generation (i.e., mitochondria and various oxidases) and its removal (i.e., catalases, peroxiredoxins, and peroxidases). However, various disease states can lead to an imbalance in H_2_O_2_ regulation, thereby causing cellular oxidative damage and toxicity.

Acute, pulsatile increases in H_2_O_2_ are viewed as largely beneficial as they typically coordinate intracellular signal cascades via disulfide switches and redox nodes. Chronic elevation, however, will cause long term changes in the redox environment and disrupt homeostasis. Major targets of H_2_O_2_ signaling are mitogen-activated protein kinases (MAPKs). H_2_O_2_-dependent signaling changes to these regulatory enzymes are known to impair cellular glucose uptake, thus contributing to onset and progression of diabetes as well as neurological disorders [65, 66]. These signaling pathways can also induce pro-inflammatory and pro-apoptotic mechanisms, the over activation of which are known to cause neurodegeneration, cardiovascular, and renal dysfunction [63, 64, 67]. Alternatively, cancer cells have an adapted redox balance that uses H_2_O_2_ signaling to promote cell proliferation through activation of PI3K/MAPK pathways. This signaling is combined with H_2_O_2_’s activation of the antioxidant regulator NF-kB to maintain cell viability despite the increased oxidative stress experienced during tumor growth [68, 69]. Another important target of H_2_O_2_ signaling includes the calmodulin-binding kinase II (CaMKII). Oxidation of CaMKII leads to its phosphorylation of downstream targets including glutamate receptors and synapsin 1 in neurons and calcium channels in cardiomyocytes. For this reason, oxidized CaMKII has been implicated to have a pathogenic role in neurological and cardiovascular disorders [70].

H_2_O_2_ is stable, can pass through biological membranes, and at most concentrations observed in cells in vivo, is not toxic. In vitro models do indicate that at high concentrations, H_2_O_2_ can induce oxidative damage to cellular proteins [71], lipids [72, 73], and DNA [74]. This is largely attributed to H_2_O_2_’s ability to react with Fe^2+^ and generate the highly reactive hydroxyl radical (·OH) [75, 76]. H_2_O_2_ can also react with Cu^1+^ to generate the reactive copper-peroxyl species (Cu(I)-OOH) [77]. Consequences of oxidative damage are broad and can be severe, particularly under disease states where antioxidant defenses are compromised. Protein oxidation can cause macromolecular changes such as cross-linking, unfolding, and sulfhydryl oxidation, often leading to proteasomal degradation [78]. Oxidation of nucleic acids can cause impaired gene expression and genomic instability [79]. Lipid oxidation can disrupt membrane structure and permeability, often leading to changes in membrane potential in excitable cells. Additionally, lipid peroxidation products themselves are reactive and capable of inducing their own oxidative damage [73, 80].

### NH_4_^+^

NH_4_^+^ is generated in all tissues via metabolism of nitrogen-containing compounds and is rapidly cleared due to its potential toxicity. Even small increases in NH_4_^+^ concentration can have toxic effects, frequently manifesting as severe neurological disorders. Consequently the body is well-equipped to clear the small molecule quickly via the urea cycle [81]. Because of its swift clearance, NH_4_^+^ is generally not considered a biologically relevant metabolite. However, recent investigation into NH_4_^+^ toxicity has identified several cellular processes directly impacted by the compound.

Increases in NH_4_^+^ production causes elevation in cellular pH which is known to have a number of physiological consequences [82]. Recently, Zhang, et al. showed that excessive NH_4_^+^ increased lysosomal pH in CD8^+^ T cells inhibiting lysosomal NH_4_^+^ storage. This caused NH_4_^+^ reflux into mitochondria leading to mitochondrial damage and ultimately cell death [83]. NH_4_^+^ is also known to alter membrane potential in cells. Because of their similar size and charge, extracellular NH_4_^+^ can compete with K^+^ for ion transport, allowing NH_4_^+^ to more readily enter cells and depolarize membranes [84, 85]. Studies have also shown that increased NH_4_^+^ depolarizes mitochondrial membrane potential leading to mitochondrial permeability transition (MPT) and cell death [86, 87]. Other derangements in mitochondrial metabolism have been associated with high NH_4_^+^ concentrations, specifically oxidative phosphorylation. Several studies have linked NH_4_^+^ to reduced cellular ATP levels, likely due to its ability to inhibit tricarboxylic acid (TCA) cycle and electron transport system (ETS), reflected in lower rates of respiration [88–90]. Additionally, increased oxidative stress has been observed after NH_4_^+^ treatment in various cell types [86, 91, 92]. This is typically attributed to increased nitric oxide (NO) production [93, 94]. NH_4_^+^-induced oxidative stress is known to participate in MPT opening and stimulate inflammation through MAPK activation.

### Catecholaldehydes

The catecholaldehydes DOPAL and DOPEGAL are MAO-generated metabolites of DA and NE, respectively. The combination of catechol and aldehyde moieties render these molecules highly reactive, and while concern over their toxicity was suggested as far back as 1952 [95], early studies concluded that these metabolites were inactive and of little consequence [96]. This notion was reinforced by the discovery of detoxification pathways that quickly metabolize catecholaldehydes into less reactive acids and alcohols via aldehyde dehydrogenase (ALDH) and aldehyde reductase (AR) [6]. For this reason, catecholaldehyde formation by MAO was largely ignored by researchers and did not emerge again in the literature until the 1990 s when DOPAL detection was reported in post-mortem substantia nigra tissue of PD patients but not in control patients [97]. Additionally, these studies showed that DOPAL reacts with numerous protein targets in the brain and elicit cytotoxic effects in neuronal cell lines [97, 98]. These findings facilitated entirely new theories concerning PD pathogenesis and eventually led to the Catecholaldehyde Hypothesis which posits that the buildup of these aldehyde metabolites is a significant contributor to the pathogenesis of neurodegenerative diseases [6]. Work supporting this hypothesis determined that DOPAL treatment increased oxidative stress and induced neuronal toxicity [99–101]. Additional research has identified mitochondria as major targets of catecholaldehydes, likely owing to the fact that MAO is anchored to the outer mitochondrial membrane. Multiple studies have connected DOPAL production to impaired mitochondrial respiration and OxPHOS, depolarization of mitochondrial membrane potential, and increased MPT-induced apoptosis [102–105].

Catecholaldehydes mediate their cellular toxicity not only through oxidation of biomolecules, but also via their covalent modification. Importantly, catecholaldehydes are capable of protein crosslinking due to their catechol and aldehyde moieties [106]. This not only leads to protein dysfunction and ultimate degradation, but can also cause protein aggregation. Exciting recent studies have shown that DOP(EG)AL conjugation to tau and α-synuclein increases their aggregation in the brain and this contributes to Alzheimer’s and PD pathogenesis [107–109], although the exact mechanisms of this DOP(EG)AL-mediated aggregation are not known. Similar to H_2_O_2_, catecholaldehydes have the ability to stimulate additional ROS in a vicious cycle. DOPAL can react with H_2_O_2_ generating toxic hydroxyl radicals, and the catechol groups on DOP(EG)AL can auto-oxidize forming reactive semi-quinone radicals and ortho-quinones [110, 111]. Generation of these additional ROS are thought to exacerbate the oxidative damage caused by catecholaldehydes in cells and tissues.

## Pathophysiologic Mechanisms Linking Catecholamine Metabolites to Heart Disease

Given the clear evidence that MAO activity is pathogenic and contributes to cardiac injury/disease, obvious questions remain concerning the mechanisms by which this occurs. The preceding section has outlined ways all three catecholamine metabolites are biologically active and potentially toxic. Below, we will review and discuss recent studies that support a mechanistic role for these metabolites in the pathogenesis of heart disease.

Nearly all reports concerning the role of MAO in cardiac disorders have documented MAO-dependent increases in ROS and oxidative damage in the heart. These studies employed a wide variety of ROS-sensitive fluorogenic probes including dihydroethidium, MitoSOX, and Amplex Red, and have consistently reported similar findings—namely, that MAO-dependent increases in H_2_O_2_ and O_2_^·−^ always occur in the heart under varying degrees of stress and disease [42, 47, 50, 58, 59]. One study used the trapping agent 4-hydroxybenzoic acid to measure ·OH production during I/R injury and observed that formation of its hydroxylated metabolite, 3,4-dihydroxybenzoic acid, was attenuated with pargyline treatment, implicating MAO as the source of the oxidation. Markers of oxidative damage accompany the increases in MAO-generated ROS. Indeed, lipid peroxidation products [56], protein carbonylation [34, 43, 50], and DNA oxidation [112, 113] are all increased in various cardiac disease states and are mitigated with MAO-A/B inhibition. Pathophysiologic effects resulting from this oxidative damage have recently been identified through mechanistic studies.

One such study observed MAO-dependent Ca^2+^ mishandling in an I/R mouse model. They identified VDAC and MCU as protein targets for MAO-A-generated oxidative stress, leading to mitochondrial Ca^2+^ accumulation, impaired respiration and loss of membrane potential [114]. Other work in humans with ischemic heart disease found elevated oxidative stress markers and actin oxidation in the ventricles of patients with heart failure. This oxidative damage in the ventricles was determined to be strongly associated with MAO-A activity and a decline in cardiac functional parameters. These findings suggest MAO-dependent oxidation of actin may impair sarcomere function, thereby contributing to heart failure [34].

Other work has suggested that MAO-dependent oxidative damage creates its own vicious cycle leading to more MAO-dependent oxidative stress. Work in neuronal cells found that ALDH and AR are inhibited by the lipid peroxidation products 4-HNE and MDA, although they have no impact on MAO activity [115, 116]. This indicates that oxidative damage generated by MAO can impair the primary enzymes responsible for catecholaldehyde detoxification, creating a bottleneck effect and exacerbating MAO-dependent toxicity. This toxicity will be exacerbated under conditions where antioxidant defenses have been compromised. For example, several studies have reported that ALDH activity is reduced in cardiac tissue of diabetes patients, which further supports the concept of a detoxification bottleneck [54, 117].

MAO activity has also been shown to alter cardiomyocyte redox signaling in a number of studies. Work by Shi and colleagues linked cardiomyocyte MAO-A to catecholamine-induced arrhythmogenesis and identified oxidation of CaMKII and PKA as part of the mechanism. This study further showed that MAO-dependent ROS in cardiomyocytes after epinephrine stimulation causes oxidative modification and phosphorylation of Ca^2+^ channels such as ryanodine receptor, thereby disrupting Ca^2+^ handling and increasing risk for arrhythmia. Another group found that nuclear translocation of the autophagy and lysosome regulator transcription factor-EB (TFEB) was blocked in the hearts of mice with cardiomyocyte-specific MAO-A overexpression. This was associated with activated mTORC1, a TFEB inhibitor, as well as lysosomal dysfunction, impaired autophagy, and mitochondrial fragmentation [118]. The authors interpreted these findings to indicate that MAO-dependent oxidative stress stimulates mTORC1, leading to autophagy dysregulation and subsequent heart failure. Another study by the same group using that model found that cardiac MAO-A overexpression led to a dilated cardiomyopathy and significant cardiac mitochondrial damage and cardiomyocyte necrosis. They attributed the MAO effect to be driven by oxidative stress-mediated activation of p53 and down regulation of PGC-1α, a key mitochondrial regulator, leading to mitochondrial dysfunction and cell death [112].

Only a few studies to date have attempted to provide insights into catecholaldehyde-mediated toxicity from MAO activation in the heart. A recent study showed that NE stimulated pro-fibrotic and pro-inflammatory signaling in murine cardiac fibroblasts, including synthesis and secretion of collagens-1 and -3, and that this effect was mediated by MAO [119]. This stimulatory effect of NE in the fibroblasts was mirrored by treatment of these cells with DOPEGAL-modified albumin, and the effect was shown to be mediated by activation of the receptor for advanced glycation end products (RAGE). More importantly, both NE and DOPEGAL-albumin effects in the fibroblasts were mitigated by co-incubation with L-carnosine, a potent aldehyde scavenger. Work by this same group has documented the presence of catechol-modified proteins and MAO-mediated catecholaldehyde formation in mitochondria from human myocardium. In one report, investigators found that DOPAL attenuated ADP-stimulated respiration in isolated mitochondria prepared from human atrial tissue samples, and that co-incubation of the mitochondria with L-carnosine blocked the DOPAL effect [120]. Another study using atrial appendage samples obtained from patients undergoing elective cardiac surgery found elevated levels of catechol-modified proteins in myocardial tissue from patients with diabetes compared with age-matched non-diabetes patients. Further proteomic analysis of these atrial tissues identified > 300 catechol-modified proteins in mitochondria, including ALDH2 and many ETC subunits and ATP synthesome components [54]. Using permeabilized atrial myofibers prepared from these samples, investigators also found that NE incubation disrupted mitochondrial OxPHOS in a MAO-dependent manner and this effect was enhanced in the samples from diabetes patients, suggesting a possible link between catechol protein modifications and altered mitochondrial function. Although the collective body of work regarding catecholaldehyde-mediated toxicity in the heart remains sparse, research in this area will likely increase given the recent findings concerning catecholaldehydes and neurodegeneration discussed above [107, 108].

## Therapeutic Strategies to Target MAO in Heart

**Fig. 2:**
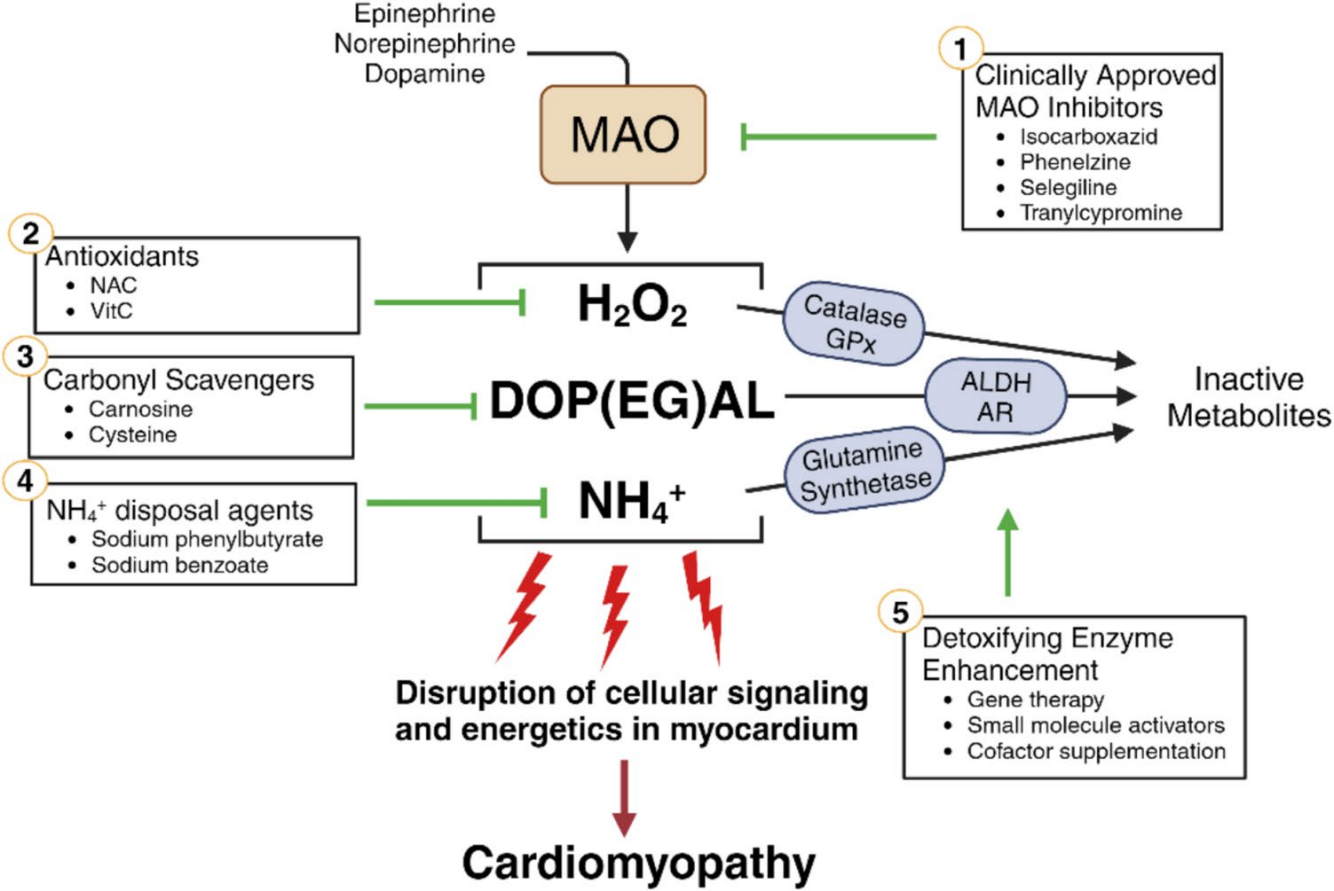
Therapeutic targeting strategies. Clinically applicable strategies for mitigating MAO-mediated toxicity at the enzyme and metabolite level are shown. (1) FDA-approved MAO inhibitors; (2) Antioxidant and (3) carbonyl scavengers; (4) NH_4_^+^ disposal agents; (5) Increased activity of phase II detoxifying enzymes. ALDH, aldehyde dehydrogenase; AR, aldoketo-reductase; GPx, glutathione peroxidase; GSH, glutathione; NAC, N-acetylcysteine. Created using bioRender.com

Owing to its rich pharmacological research history, there are numerous small molecule MAOI’s that have been developed and studied, and, while not all are clinically approved, many of them have shown therapeutic potential in experimental models of cardiac injury and disease. Mechanistic studies described in previous sections have also clearly shown that metabolites formed by MAO are biologically active, therefore targeting these metabolites may be a therapeutically effective option, either alone or in combination with MAO inhibition. This section discusses viable strategies for targeting MAO and its metabolites, many of which are already in clinical use (Fig. 2).

Use of MAOIs in the treatment of psychological disorders have been well-documented in the medical and scientific literature for > 60 years [121]. Current FDA-approved MAOIs include isocarboxazid, phenelzine, selegiline, and tranylcypromine, and are used to treat depression, anxiety, and PD. Interestingly, several studies have reported cardiovascular benefits of these drugs. Early clinical studies of MAOIs in patients with mood disorders reported their anti-hypertensive [122, 123] and anti-anginal effects [124]. Our group recently found that patients taking MAOIs for clinical depression had lower risk for arrhythmogenesis compared to patients taking SSRIs [50]. These studies justify a repurposing of MAOIs for the treatment of cardiac injury/disease in both restorative and preventative context. However, an important factor to consider with current clinically approved MAOIs is that they all have strong centrally mediated sympathomimetic effects. While this dual action between the heart and the brain may be useful for treating both comorbidities of MAO-associated diseases [125], unwanted neurological effects can also be prevented by small modifications to reduce blood–brain transport of the MAOI.

An altogether different strategy that may prove to be more effective at mitigating cardiac pathophysiology would be to target the products of MAO-mediated catecholamine metabolism, rather than the enzyme itself. In this way, catecholamine levels would not be altered and the side effects and interactions of MAOIs could be largely avoided. It is well known that enhancing antioxidant enzymes, specifically catalase and glutathione peroxidases (GPx’s) which reduce H_2_O_2_ to H_2_O, is a very effective strategy to mitigate oxidative stress [63]. Studies have shown that increased GPx [126, 127] and catalase [128–130] activity improve cardiac electromechanical function and reduce ROS toxicity in animal models that have undergone IR injury, doxorubicin-induced cardiomyopathy, diabetes, and heart failure. In addition to gene therapy (which is yet to be realized as a viable clinical option for heart disease), a strategy for enhancing antioxidant enzymes in heart can include vitamin/cofactor supplementation and clinically approved drugs that upregulate the antioxidant response system in the heart (e.g., Nrf2, PPAR-γ activators) [131]. Direct administration of antioxidants (e.g., vitamin C) can also enhance ROS detoxification [132]. N-acetylcysteine (NAC) is also a strong antioxidant due to its glutathione replenishing effect [133]. Recent studies have demonstrated the cardioprotective properties of NAC in a number of ROS-related cardiac disorders [134, 135]. Its strong sulfur smell and nausea-inducing effects limit its utility in the clinic, however, where it has been most commonly employed as a mucolytic agent for cystic fibrosis. Natural medicines (e.g., Terminalia arjuna) have also been shown to benefit the heart through antioxidant and anti-inflammatory properties [136].

While its role in mediating effects of MAO activity in the heart remain unclear, NH_4_^+^ is also a product of catecholamine metabolism that can be disposed of by targeting the body’s natural NH_4_^+^ removal processes such as urea cycle and glutamine synthesis enzymes [81]. Alternative metabolic routes for NH_4_^+^ removal include the use of sodium benzoate and phenylbutyrate, which form conjugates with glycine and glutamine, respectively, that are then excreted from the body [137, 138]. These therapies are typically applied in the context of neurological disorders and liver dysfunction and consequently their effects on the heart are not well known. However, recent studies have found that phenylbutyrate treatment improved cardiac function in experimental models of pressure overload, IR, and doxorubicin-induced cardiomyopathy [139–142]. Although the therapeutic effect of phenylbutyrate was attributed by the authors to be due to alleviation of ER stress, the mechanism of action remains incompletely understood and provides some justification for targeting NH_4_^+^ removal as therapy for heart disease.

Catecholaldehydes are another intriguing product of MAO-mediated catecholamine metabolism that could be therapeutically targeted to mitigate heart disease. One strategy may be to increase phase II detoxification enzymes ALDH and AR in heart, thereby facilitating catecholaldehyde neutralization to less reactive carboxylic acids and alcohols. The small molecule Alda-1 has become a point of interest in this regard because of its ability to activate ALDH2 and enhance aldehyde detoxification [143–145]. Studies have demonstrated that Alda-1 treatment in animal models protects against myocardial injury with reduced infarct size, mitigated contractile dysfunction, and improved mitochondrial function [146–148]. Direct scavengers of catecholaldehydes have also been identified and may act as another therapeutic option. Research has shown that the amino acids l-carnosine (B-alanyl-l-histidine) and l-cysteine readily react with DOP(EG)AL, forming stable conjugates that are then targeted for degradation [120, 149]. These amino acids are endogenous, completely non-toxic and, while there are challenges to their pharmaceutical delivery, their supplementation remains a viable option for neutralizing catecholaldehyde toxicity in the heart.

## Conclusions and Unresolved Questions in the Field

While evidence supporting a pathogenic role for MAO in cardiac disease has continued to emerge, new and old questions related to causation persist. A major challenge to the field is consistent and reproducible methods to detect and measure catecholaldehydes in biological material. The labile and highly reactive nature of these molecules renders them almost undetectable as free aldehydes, even with the most sensitive LC–MS methods. As discussed above, recent advancements in molecular detection by our group and others have enabled measurement of stable DOP(EG)AL conjugates [119, 149] and catechol protein modification [54, 107, 108] in cells and tissues. However the extent to which these modifications contribute to disruptions in cellular homeostasis and disease pathogenesis remains unknown. Specifically, it is unclear whether they are just indiscriminate markers of oxidative damage, or whether discrete modifications on certain targets disrupt homeostasis in the cell, and by what mechanism this is occurring. Likely this will require improvements in biochemical analysis to detect DOP(EG)AL modification at specific sites within proteins and other macromolecules, combined with innovative methods to determine how these modifications are disruptive, if at all. There also remains a strong possibility that DOP(EG)AL may interact directly with neurotransmitter signaling, particularly adrenergic. An interesting study using the β1 adrenergic agonist isoproterenol to induce cardiomyopathy in rodent models showed that the β1 receptor and RAGE structurally and functionally interact to induce cardiomyocyte hypertrophy and cell death [150]. Given that DOPEGAL-modified proteins serve as ligands of RAGE as discussed in the study above [119], it is plausible that there are synergistic effects driven by catecholaldehydes and catecholaminergic/serotonergic signaling via this β1R/RAGE crosstalk.

In addition to mechanisms of catecholaldehyde toxicity, other important questions pertain to the mitochondrial specific effects of MAO-mediated catecholamine metabolism in vivo. Since most of the experimental studies linking mitochondrial abnormalities to MAO have used in vitro models with supraphysiological concentrations of catecholamines, it will be important for investigators to ascertain whether these effects are happening in the heart itself and to what extent these effects change over time. In this context, outstanding questions remain concerning the NH_4_^+^ formed via MAO-mediated catecholamine metabolism in the mitochondrial inner membrane space where it could readily participate in aminotransferase reactions and anabolic protein synthesis. Future studies are clearly needed to investigate these questions more thoroughly.

To conclude, it is clear from the studies discussed above that products formed via MAO-mediated catecholamine metabolism contribute to acute and chronic diseases, particularly in the heart. For this reason, we hope that clinician scientists, medical instructors, and pharmacology textbook authors will gain more appreciation for the impact of MAO activity, especially given that these metabolites are ‘not inactive after all.’ Indeed, they are very biologically active and most importantly, they can be therapeutically targeted.

## Data Availability

No datasets were generated or analyzed during the current study.
